# The indication and curative effect of hysteroscopic and laparoscopic myomectomy for type II submucous myomas

**DOI:** 10.1186/s12893-016-0124-7

**Published:** 2016-02-27

**Authors:** Haibo Wang, Jinrong Zhao, Xiujuan Li, Ping Li, Caihong Lu, Shujuan Tian, Zhong-hua Wang

**Affiliations:** Department of Obstetrics and Gynaecology, The 260th Hospital of PLA, No. 346 Shengli North Street, Shijiazhuang, 050041 China

**Keywords:** Submucous myomas, Fibroids, Myomectomy, Hysteroscopy, Laparoscopy

## Abstract

**Background:**

The aim of this study was to assess curative effect of hysteroscopic and laparoscopic myomectomy for type II submucous myomas between 3 and 5 cm in diameter and explore the optimal surgical indications.

**Methods:**

A retrospective analysis was performed of those who underwent hysteroscopic or laparoscopic myomectomy from January 2008 to January 2013. The patients were divided into three subgroups according to the myomas diameter (namely, 30 mm ≤ myomas diameter <40 mm; 40 mm ≤ myomas diameter <50 mm; and myomas diameter ≥ 50 mm). Clinical data such as operation time, amount of bleeding, postoperative anal exsufflation time, hospital stay, and complications were collected.

**Results:**

There was no significant difference regarding operation time and amount of bleeding in two groups. We found significant difference in hysteroscopic group (within-subgroup) difference regarding operation time and amount of bleeding, whereas no significant difference in the laparoscopic group, while significant differences between-subgroup differences regarding operation time. Complete removal of myoma was seen in all patients.

**Conclusions:**

Both techniques are feasible for type II submucous myomas. Laparoscopic operation has higher advantages in type II submucous myomas of greater than 4 cm in diameter whereas hysteroscopic operation has higher advantages in type II submucous myomas of lower than 4 cm in diameter.

## Background

Uterine myomas are the most common benign tumors of the female genital tract affecting approximately 30 % of women by the age of 35 and 70–80 % of women aged ≥ 50 [1, 2]. Depending on their location in the uterus, they may be subserous, intramural, or submucous. Submucous myomas account for 5.5 % to 10 % of all uterine myomas [3]. Submucous myomas are sub-classified into type 0, I and II depending on the degree of intramural involvement. Type 0 myoma is entirely in the endometrial cavity, type I myoma extends less than 50 % into the myometrium and type II myoma extends greater than 50 % into the myometrium [4, 5]. It may induce severe clinical symptoms such as abnormal menstrual bleeding, infertility, pelvic pain, and dysmenorrhea [6–8].

A variety of therapeutic options are available for submucous myomas, such as myomectomy (using laparotomic, laparoscopic or hysteroscopic access), subtotal or total hysterectomy (by laparotomy or laparoscopy). With the popularization of the concept of the minimal-access surgical techniques, hysteroscopic and laparoscopic operations have predominated in recent years [9–11]. Currently, it is possible to treat symptomatic submucosal myomas less invasively, with good results, with hysteroscopic myomectomy [5, 12–15]. However, the current literature has revealed some limits of the hysteroscopic approach to treating submucous myomas such as the size, location, and position of the myoma [13, 16] and associated with risks such as longer operation time, incomplete removal of myomas, and uterine perforation. Laparoscopic myomectomy (LM) is considered one of the major breakthroughs because of the proven advantages with respect to post-operative pain and the shorter hospitalization and convalescence and for the cosmetic reasons [17]. However, it involves the repair of myometrium and removal of myoma from the abdomen and thus it requires more technical skill than many other laparoscopic procedures. Hence, it is a debated surgical procedure for laparoscopic physicians and is relatively time-consuming that may induce greater blood loss compared to the traditional open operations [18–20]. But with the improvement of surgical skills, the indications of laparoscopic myomectomy have broadened gradually, especially for large submucous myoma. In this study, we assessed the clinical efficacy and safety of hysteroscopic and laparoscopic myomectomy for submucous myomas between 3 and 5 cm in diameter. In addition, the optimal indications of these two surgical approaches for submucous myomas were also discussed.

## Methods

### Patients

The study was approved by the institutional review board of the 260th Hospital of PLA and conducted in accordance with the Declaration of Helsinki. Written informed consent was obtained from each patient. We retrospectively analyzed the clinical data of patients with type II submucous myomas between 3 and 5 cm in diameter treated with hysteroscopic or laparoscopic myomectomy at our hospital from January 2008 to January 2013. Type II submucous myomas in this study was sessile with an intramural extension of at least 50 % [21]. The diagnosis of type II submucous myomas in this study was determined by ultrasound and hysteroscopic examination according to the European Society of Hysteroscopy classification [21, 22].

The inclusion criteria was as follows: (1) patients with menorrhagia, secondary anemia, and infertility; (2) single type II submucosal myomas s with diameter between 3 and 5 cm; (3) the free myometrial margin (FMM) had to be at least 2 mm; (4) patients with uteri of less than 10 weeks and uterine cavity depth ≤ 12 cm; (5) preoperative examination showing uterine fibroid without endometrial lesions; (6) Thinprep cytology test (TCT) showing no malignant lesions of the uterine cervix.

The exclusion criteria was shown as follows: (1) patients who are contraindicated to hysteroscopic and laparoscopic operation; (2) patients who fail to achieve adequate cervical dilatation due to cervical scar; (3) patients who cannot undergo hysteroscope placement due to minimal angle between uterine body and cervix uterus; (4) reproductive tract infections at acute phase; (5) Type I submucous myomas with or without sessile; (6) subserous myoma; (7) uterine adenomyoma; and (8) a FMM of 2 mm or lower.

### Operative technique

All patients underwent preoperative assessment, including detailed medical history, pelvic examination, ultrasound examinations with the use of transvaginal probes (3.5 ~ 7.0 MHz), and diagnostic hysteroscopy using a 2.9-mm 30° rod lens hysteroscope (26120 BA; Karl Storz, Tuttlingen, Germany). All procedures were performed by a senior endoscopic physician and a skillful assistant.

### Hysteroscopic myomectomy

Under combined spinal-epidural anesthesia, the operation was performed with the patient placed in the lithotomy position. Under the monitoring of the location of the myomas and uterine intramural wall, the cervix was dilated up to a size of 12-mm with metal dilator. An operative hysteroscope (Karl Storz, 140 Tuttlingen, Germany) with a 12 mm external diameter was inserted. The uterus cavity was distended by 5 % dextrose in normal saline solution installed by automatic fluid pump (Endomat, Karl Storz, Tuttlingen, Germany). After detecting protuberance region or white region of the mucosa by hysteroscope and ultrasound images, the protuberance region or white region of the mucosa parallel to the uterine wall was dissected and then removed with a 90-degree loop electrode (cutting power, 80 W; electrocoagulation power, 60 W). After exposing the myomas nucleus following the dissection of the myomas endometrium, the application of electric knife should be positioned on the myomas nucleus avoiding the injury to the surrounding muscular layer around the myomas. For type II submucous myomas with myomas diameter of greater than 4 cm, oxytocin was intravenous injected with a maximal usage of 30 units. Electric knife stimulation and the application of oxytocin can induce uterine contraction and extrude the myomas nucleus to uterine cavity. Under this condition, edge cutting of the myomas nucleus that protruding into the uterine cavity was performed using electric knife. After most part of myomas nucleus was removed, the middle long narrow part (residual part) of the myomas nucleus was kept. Then the residual myomas nucleus which has protruded into the uterine cavity was clipped out using a toothed ring forceps and pulled out from myomas pseudocapsule bluntly and finally was resected completely. After surgery, “O” type (inert metals) intrauterine device was placed on the uterine cavity. Estradiol Valerate (Bayer Schering Pharma AG, Berlin, Germany) was administered orally on a fixed postoperative dosing schedule 3 mg q12h for 21 days. If there exists breakthrough bleeding, 3 courses of sequential therapy was administered to promote the uterine endometrium growth consisting of oral medroxyprogesterone acetate (Zhejiang Xianju Pharmacy Ltd, Zhejiang, China) at 6 mg once daily for 10 days. The location of the myoma and uterus wall was monitored with B-mode ultrasound throughout the whole course of procedure. Patients who had large amount of bleeding underwent uterine compression with an intrauterine Bakri balloon (Cook Medical, Bloomington, IN).

### Laparoscopic myomectomy

The surgeries were performed under general anesthesia with the patient in head down and lithotomy position. Veress needle is inserted periumbilically to establish an artificial pneumoperitoneum. With a pneumoperitoneum pressure of 12–14 mm Hg, a 10-mm trocar was placed in the upper edge of umbilicus after removal of the Veress needle. Video-laparoscopy was introduced after inspection of the abdominal cavity. We used three accessory ports: the first and second ports were inserted from the left side of the abdomen (15 mm and 5 mm), and the third port inserted from the right side of the abdomen (5 mm). According to preoperative ultrasonic positioning findings, the location of the submucous fibroid was determined at laparoscopy. 6 μ diluted hypophysin was injected into the intramural wall. A unipolar electrode was used to dissect the superficial muscular layer overlying the myoma. The incision was extended to the surface of the tumor nucleus. The capsule overlying myoma was separated bilaterally carefully. The grasping forceps were used to enucleate the myoma by gentle traction and countertraction until the myoma was enucleated completely. Attention should be paid while enucleating the myoma to prevent damage to the underlying endometrium. Myoma capsule protruding into the uterine cavity was dissected bluntly. When enucleating the myomas completely, the endometrium will protrude into the tumor cavity. In case that the endometrium was damaged, it was closed with 2–0 Vicryl (Johnson & Johnson, New Brunswick, New Jersey). The superficial myometrium is closed with an interrupted absorbable layer of 1–0 Vicryl (Johnson & Johnson, New Brunswick, New Jersey). Serosal layer is sutured with 3–0 continuous absorbable subcuticular sutures. All procedures were video monitored, and all resected specimens were sent for histologic analysis.

### Follow up and assessment index

The patients were followed up at 1, 3, 6 months and thereafter every 6 months postoperatively. The follow up contents included the conditions of menstruation, abdominal pain, secondary dysmenorrhea, etc. Transvaginal ultrasonography examination was conducted to inquire myometrium echo and whether there exists myomas recurrence. For patients whose endometrium was damaged during hysteroscopic or laparoscopic operations, hysteroscopic examinations were used to inquire the morphology of the uterine cavity and if there exists scars or polypus on the primary myomas location after operation.

In this study, the following parameters were observed operation time, the amount of bleeding during operation, postoperative anal exsufflation time, postoperative recurrence, the days of hospital stay and all potential possible complications such as trans urethral resection of prostate (TURP) syndrome, infection, uterine perforation, omental emphysema, mesenteric contusion, intrauterine adhesion, secondary dysmenorrhea, and adenomyosis.

Operating time of the hysteroscopic operation was defined from the initial insertion of the hysteroscopy until the operation is over. Operating time of the laparoscopic operation was defined from the initial incision of the skin until the operation is over. The amount of bleeding during hysteroscopic operation was estimated using following equation: (preoperative hematocrit (HCT)-intraoperative HCT)/(preoperative HCT × Weight (Kg) × 7 %). The amount of bleeding during laparoscopic operation was measured volume of the suction fluid minus applied flushing fluid.

### Statistical analysis

All data were analyzed using SPSS16.6 statistical software (SPSS Inc., Chicago, IL). Categorical data was expressed as frequency or percentage and compared with *χ*2 test. Continuous data was expressed as the mean ± standard deviation and compared using *t* test or analysis of variance as appropriate. A *P* value of less than 0.05 was considered statistically significant.

## Results

A total of 85 patients with type II submucous myomas between 3 and 5 cm in diameter treated with hysteroscopic or laparoscopic myomectomy. Of the 85 patients with type II submucous myomas, 40 cases were treated with hysteroscopic myomectomy (hysteroscopic group) while the remaining 45 cases were treated with laparoscopic myomectomy (laparoscopic group). The mean age of patients in hysteroscopic group and laparoscopic group was 32.62 ± 11.42 years old and 33.41 ± 10.51 years old, respectively. The mean myoma diameter of patients in hysteroscopic group and laparoscopic group were 4.04 ± 0.96 cm and 4.13 ± 0.87 cm, respectively. There was no statistically significant difference regarding to the age, BMI, parity, fibroid diameter and comorbidities between the hysteroscopic and laparoscopic groups (Table 1).

**Table 1:** Baseline data in two groups

Index		Hysteroscopic group (*n* = 40)	Laparoscopic group (*n* = 45)	*P* values
Age(years)		32.62 ± 11.42	33.41 ± 10.51	0.74
BMI		23.66 ± 3.58	23.41 ± 3.38	0.74
Parity				
Nulliparous		6	7	0.94
Parous		34	38	0.94
Myoma diameters (cm)		4.04 ± 0.96	4.13 ± 0.87	0.65
Comorbidity				
Diabetes		9 (25.50 %)	12 (26.67 %)	0.66
Hypertension		8 (20.00 %)	12 (26.67 %)	0.47

*BMI* Body Mass Index

### Perioperative data

Perioperative data in the hysteroscopic and laparoscopic group was reported in Table 2. There was no statistically significant difference regarding the operation time and amount of bleeding during operation between the two groups. The postoperative anal exsufflation time was significantly higher in the laparoscopic group (22.41 ± 4.25 h) than the hysteroscopic group (11.65 ± 3.21 h, *P* < 0.01). The hospital stays was significantly higher in the laparoscopic group (5.56 ± 1.24 days) than the hysteroscopic group (3.23 ± 1.65 days, *P* < 0.01). When the patients in the hysteroscopic and laparoscopic group were further divided into three different subgroups according to myomas diameter (namely, Group A, 30 mm ≤ myomas diameter <40 mm; Group B, 40 mm ≤ myomas diameter <50 mm; and Group C, myomas diameter ≥ 50 mm), we found statistically significant within-subgroup differences regarding operation time and amount of bleeding in the hysteroscopic group (*P* < 0.05) and no statistically significant difference in above parameters (including operation time, amount of bleeding, postoperative anal exsufflation time, and hospital stays) in the laparoscopic group (all *P* >0.05). Regarding between-subgroup comparisons, we observed statistically significant differences in operation time between all three subgroups (Group A, B, and C) and the amount of bleeding between two subgroups (Group B, and C). Perioperative data in the different subgroups among hysteroscopic and laparoscopic group were shown in Table 3.

**Table 2:** Perioperative data in the hysteroscopic and laparoscopic group

	Hysteroscopic group (*n* = 40)	Laparoscopic group (*n* = 45)	*P* values
Operation time (min)	56.78 ± 16.48	62.21 ± 20.12	0.18
Amount of bleeding during operation (ml)	78.25 ± 35.18	85.87 ± 20.23	0.22
Postoperative anal exsufflation time (h)	11.65 ± 3.12	22.41 ± 4.25	0.00
Hospital stays (days)	3.23 ± 1.65	5.56 ± 1.24	0.00

**Table 3:** Perioperative data in the different subgroups among hysteroscopic and laparoscopic group

	Operation time (min)	Amount of bleeding during operation (ml)	Postoperative anal exsufflation time (h)	Hospital stays (days)
Hysteroscopic group (*n* = 40)				
Group A (*n* = 19)	45.66 ± 13.31	42.23 ± 23.21	11.21 ± 1.87	3.12 ± 1.20
Group B (*n* = 15)	65.67 ± 20.32	95.54 ± 33.32	10.87 ± 1.56	3.23 ± 1.11
Group C (*n* = 6)	76.24 ± 16.73	105.25 ± 32.87	11.22 ± 2.15	3.45 ± 1.42
*P* values	0.0003	0.0000	0.8449	0.8035
Laparoscopic group (*n* = 45)				
Group A (*n* = 27)	59.46 ± 18.23^a^	64.65 ± 25.33	21.23 ± 1.65	5.10 ± 1.20
Group B (*n* = 12)	56.74 ± 10.23^a^	74.47 ± 44.78^a^	21.34 ± 1.76	5.43 ± 1.19
Group C (*n* = 6)	62.46 ± 10.65^a^	76.43 ± 42.54^a^	22.42 ± 1.34	5.33 ± 1.47
*P* values	0.7573	0.5940	0.2831	0.8202

^a^,compared to hysteroscopic group; Group A, 30 mm ≤ myomas diameter <40 mm; Group B, 40 mm ≤ myomas diameter <50 mm; Group C, myomas diameter ≥ 50 mm

Three patients in the hysteroscopic group required a second surgery. Of these, two patients with a myomas diameter of 50 mm underwent second hysteroscopic operation 2 months after the operation. The remaining patient with a myomas diameter of 38 mm underwent second hysteroscopic operation 1 month after the operation. Finally the myomas were completely removed. Complete removal of myoma was observed in all of the patients in the laparoscopic group. Histologic examination of the resected tissue showed benign uterine myomas in all patients.

### Postoperative outcomes

Table 4 showed the postoperative outcomes. Postoperative recurrent myomas were not seen in any patients of the two groups. None of the patient occurred TURP syndrome and uterine perforation. Among the 45 patients in laparoscopic group, 1 case occurred omental emphysema and 2 cases occurred mesenteric contusion during operation and recovered to normal after operation.

**Table 4:** Postoperative outcome of patients treated with hysteroscopic or laparoscopic myomectomy

Index	Hysteroscopic group (*n* = 40)	Laparoscopic group (*n* = 45)
Residual or recurrent myomas	0 (0 %)	0 (0 %)
Complications	5 (16.1 %)	5 (11.1 %)
Omental emphysema	0 (0 %)	1 (2.2 %)
Mesenteric contusion	0 (0 %)	2 (4.4 %)
Mild intrauterine adhesion	5 (16.1 %)	2 (4.4 %)
Secondary dysmenorrhea	0 (0 %)	0 (0 %)
Adenomyosis	0 (0 %)	0 (0 %)

One patient in laparoscopic group experienced an accidental pregnancy 14 months after the operation. The patient had a uterine scar less than 2 years which had the risk of hysterorrhexis. But she insisted on the pregnancy and finally delivered an infant following cesarean section at full term. In addition, there were 3 patients in laparoscopic group and 4 patients in hysteroscopic group had a full-term pregnancy 2 years after the operation. Five patients in hysteroscopic group occurred mild intrauterine adhesion 1 month after the operation. Of these, 2 cases achieved reduction of adhesions following uterine distention while the remaining 3 cases relieved adhesions with scissor. Two patients in laparoscopic group occurred mild intrauterine adhesion 1 month after the operation and achieved reduction of adhesions following uterine distention. None of the patient in the two groups occurred secondary dysmenorrhea, and adenomyosis.

## Discussion

Hysteroscopic myomectomy is the preferred method when fibroids are submucosal or when most of an intramural fibroid protrudes into the uterine cavity [9]. Hysteroscopic resection of submucous fibroids is considered as a simple, well-tolerated and effective procedure [23]. However, the number and the size of myomas play a crucial role in completing the hysteroscopic myomectomy [14]. For type II submucous myomas, there exist some surgical difficulties during hysteroscopic operation. Therefore, if laparoscopic resection can be used as an alternative surgical approach to remedy the limits of hysteroscopic is worthwhile exploring problem.

Rakesh et al. reported that laparoscopic myomectomy for large submucous myoma is a technically feasible procedure and suggest that it is appropriate for the experienced laparoscopic surgeons to look at the indications of the laparoscopic myomectomy [24]. In this study, we compared the clinical data of patients who underwent the laparoscopic and hysteroscopic myomectomy for type II submucous myomas equal or greater than 30 mm in diameter, and found no significant difference regarding the operation time and amount of bleeding between the two techniques. These findings suggest that these two techniques have achieved similar technical efficacy in these selected patients. However, when patients were further divided into 3 groups according to the diameter of the myomas, we found that hysteroscopic myomectomy have higher advantages in type II submucous myomas of lower than 40 mm in diameter in relation to operation time and postoperative recovery and whereas laparoscopic myomectomy had higher advantages in type II submucous myomas of equal or greater than 40 mm in diameter in relation to operation time, amount of bleeding. In addition, we found that 3 patients (myomas diameter 50 mm, 50 mm, and 38 mm, respectively) undergoing hysteroscopic myomectomy required secondary surgery and one-off surgical removal of myomas was obtained in patients receiving laparoscopic myomectomy. Our findings are partially consistent with the previous report which suggests that only the diameter greater than 3 cm in type II submucous myoma is correlated to a higher risk of a multiple procedure during the hysteroscopic myomectomy [14].

Hysteroscopic myomectomy is considered as the first-line conservative therapeutic option for the treatment of prolapsed pedunculated submucous myomas [25]. However, for patients who are not suitable for hysteroscopic surgery, laparoscopic myomectomy should be considered for surgeons with experienced surgical skills. Previous studies reported that for patients with type II submucous myomas in whom the size of the myoma was equal or greater than 40 mm, the surgeon should select the optimal surgical approach with discretion according to the patients’ conditions and its own technical level [26, 27]. The aforementioned findings in this study suggest the advantage of laparoscopic myomectomy in the type II submucous myomas equal or greater than 40 mm. The evidence for supporting the laparoscopic myomectomy for the treatment of type II submucous myomas equal or greater than 40 mm include: 1) improved accuracy of positioning of the myomas with the development of the ultrasound techniques; 2) the myomas can be detected and resected accurately at the best position with the laparoscopy or robotic-assisted laparoscopy; 3) intraoperative injection of hypophysin into the intramural wall helps to hemostasis; 4) complete removal of myomas with little bleeding and the intact of the uterine mucosa and 5) tumor cavity can be sutured well under the laparoscopy or robotic-assisted laparoscopy, with the advantages of clear view, accurate counterpoint and good healing outcomes after surgery. However, due to the limited sample of included patients and the retrospective design of the study, further studies with larger sample sizes and prospective design are required to validate our findings.

During the hysteroscopic resection of the type II submucous myomas, it can inevitably increase the injury to the uterine endometrium in order to completely resect the myomas, which will sow the seeds of intrauterine adhesions. Therefore, adequate size of type II submucous myomas is important for carrying out the hysteroscopic myomectomy [14]. In addition, attention should be paid to protect the myoma pseudocapsule, especially the uterine endometrium [28]. These can not only prevent the risk of the uterine adhesion due to large surgical scope, but lay a basis for subsequent pregnancy. Meanwhile, we also take care to protect the myometrium surrounding the myoma during the hysteroscopic myomectomy, which can also reduce the risk of intrauterine adhesions. Similarly, during the process of laparoscopic removal of submucous myomas, care should be also taken to preserve the intact of the endometrium when the myomas were resected. Blunt stripping should be conducted while enucleating the myoma adjacent to the uterine mucous layer. During the process of blunt stripping, application of energy equipment should be avoided. During the process of dissecting the serosal layer using unipolar electrode, a mode of electrocoagulation and electrosection with an electrocoagulation power of 40 W was used in this study. In case that the endometrium was damaged, the wound of the uterus was sutured in layers to ensure the accurate myometrial apposition and prevent the risk of postoperative intrauterine adhesion. Since the submucous myomectomy can inevitably cause a large resection cavity, attention should be paid while handling the residual cavity of the myometrium during the suture. In this study, interrupted “8” figure suture was used for hemostasis and running sutures was used for the residual cavity of the myometrium, which helps to tighten the sutures and hemostasis. We did not use electric coagulation hemostasis. This can prevent local cavity infection and thus avoid the risk of uterine rupture during pregnancy. In our series, we observed 16.1 % (5/40) of uterine adhesions (synechiae) in the hysteroscopic group and 4.4 % (2/45) of uterine adhesions in the laparoscopic group. We speculate these may be associated with the size of the myoma. During the hysteroscopic removal of large myoma, cutting a relatively large “window” on the uterine mucosa is needed which destroy the relatively more endometrium and thus increases the risk of the incidence of the uterine adhesions. Whereas the laparoscopic removal of myomas was performed through the uterine serosal surface which relatively preserve the endometrium and reduce the risk of the incidence of the uterine adhesions.

## Conclusions

Both techniques are feasible for type II submucous myomas of diameter between 3 and 5 cm. Laparoscopic operation had higher advantages in type II submucous myomas of greater than 4 cm in diameter whereas hysteroscopic operation higher advantages in type II submucous myomas of lower than 4 cm in diameter. Further studies with a larger number of samples are still necessary for confirming our findings.

## Abbreviations

TCT: thinprep cytology test
TURP: trans urethral resection of prostate
LM: laparoscopic myomectomy
FMM: free myometrial margin
HCT: hematocrit
